# Association Between Air Pollution and COVID‐19 Pandemic: An Investigation in Mumbai, India

**DOI:** 10.1029/2021GH000383

**Published:** 2021-07-01

**Authors:** Aparajita Chattopadhyay, Subhojit Shaw

**Affiliations:** ^1^ Department of Development Studies International Institute for Population Sciences Mumbai India

**Keywords:** COVID‐19, Mumbai, air pollution, hot spots, respiratory infection, spatial regression, India

## Abstract

Spatial hot spots of COVID‐19 infections and fatalities are observed at places exposed to high levels of air pollution across many countries. This study empirically investigates the relationship between exposure to air pollutants that is, sulfur dioxide, nitrogen dioxide, and particulate matter (SO_2_, NO_2_, and PM_10_) and COVID‐19 infection at the smallest administrative level (ward) of Mumbai City in India. The paper explores two hypotheses: COVID‐19 infection is associated with air pollution; the pollutants act as determinants of COVID‐19 deaths. Kriging is used to assess the spatial variations of air quality using pollution data, while information on COVID‐19 are retrieved from the database of Mumbai municipality. Annual average of PM_10_ in Mumbai over the past 3 years is much higher than the WHO specified standard across all wards; further, suburbs are more exposed to SO_2_, and NO_2_ pollution. Bivariate local indicator of spatial autocorrelation finds significant positive relation between pollution and COVID‐19 infected cases in certain suburban wards. Spatial Auto Regressive models suggest that COVID‐19 death in Mumbai is distinctly associated with higher exposure to NO_2_, population density and number of waste water drains. If specific pollutants along with other factors play considerable role in COVID‐19 infection, it has strong implications for any mitigation strategy development with an objective to curtail the spreading of the respiratory disease. These findings, first of its kind in India, could prove to be significant pointers toward disease alleviation and better urban living.

## Key Points

- Significant association between COVID‐19 infection with SO_2_, NO_2_, and PM_10_ reveals spatial hot spots in Mumbai
- With increase in NO_2_ there is substantial increase in COVID‐19 deaths in Mumbai
- Air quality is an important element to address COVID‐19 management and sustainable urban development

## 1 Introduction

A cluster of cases of pneumonia was reported in Wuhan, Hubei province of China in December 2019. Later, World Health Organization (WHO) in March 2020, declared that SARS‐CoV‐2 or COVID‐19 can be characterized as a pandemic considering its alarming levels of spread and severity (Huang et al., 2020; Li et al., 2020; WHO, 2020; Wu et al., 2020). A handful of literature suggests that the primary mode of infection might be through droplets and contaminated environmental surface transmitted from an infected person (Chaudhuri et al., 2020; Greenhalgh et al., 2021; Kampf et al., 2020). Outbreak of this disease was observed in highly polluted cities of China, Italy, Spain, UK, USA and India (Bashir et al., 2020; Fattorini & Regoli, 2020; Gupta et al., 2021; Zhu et al., 2020). European Respiratory Review stated that both short and long‐term exposure to air pollution may be important aggravating factor for SARS‐CoV‐2 transmission and lethality through multiple mechanism (Gupta et al., 2021). Further, many researchers found that the atmospheric pollutant has made a major contribution in the spread and severity of COVID‐19 infection across major cities of the world (Berman & Ebisu, 2020; Wu et al., 2020; Zhu et al., 2020).

The adverse effect of air pollution induced mortality is well recognized (Balakrishnan et al., 2019; Dholakia et al., 2014; Karuppasamy et al., 2020). Long‐term exposure to pollutants like CO, SO_2_, NO_2_, O_3_, particulate matters etc., lead to lung inflammation and respiratory diseases (WHO, 2003). One of the major transportation exhaust, NO_2_ is responsible for Chronic Obstructive Pulmonary Disease (COPD) and asthma (Baylon et al., 2018). Similarly, with deteriorating air quality and higher ultraviolet radiation (UV), the chances of lung infection response to any viral infection increases (Gerba et al., 2002; Tseng & Li, 2007). Particulate Matter like PM_10_, PM_2.5_, and aerosol also supports a platform for virus transmission (Diffey, 1991; Gerba, 1984; Manoj et al., 2020; Mi et al., 2019; WHO, 2020). Literature further indicates that hydrophilic compound absorbs humidity, bacteria, and RNA rich virus (Valsaraj, 2009). Thus, air quality plays a complex role in the transmission of respiratory infection like coronavirus (Manoj et al., 2020). Though health effects attributable to short‐term and long‐term ambient air pollution (AAP) exposure among Indian population are less understood, a meta‐analysis considering literature from 1990 to 2020, revealed statistically significant associations between ambient air pollution exposure and increased COPD, respiratory illnesses, higher rates of hospital admission as well as premature mortality in India (Rajak & Chattopadhyay, 2020).

Air pollution has considerably worsened the COVID‐19 outbreak in the US (Khadka, 2020). Among people who have lived with polluted air for decades, air pollutant particles may be acting as vehicles for viral transmission (Gerretsen, 2020). An increase of only 1 μg/m^3^ in PM_2.5_ has shown to result in 8% increase in the COVID‐19 death rate, as estimated by Xiao et al. (2020). Air pollution weakens the immune system and thus induces possibility of catching any infection like COVID‐19 (Cui et al., 2003; Glencross et al., 2020). Although air quality varies with weather conditions (Bashir et al., 2020; Liu et al., 2020; Tosepu et al., 2020), the major cities in India like Delhi, Mumbai, Bengaluru experience poor air quality throughout the year and are worst‐hit in COVID‐19 (IQAir, 2020). However, no major study has yet been conducted in Indian cities due to data constraint in obtaining pollution and COVID‐19 daily updates at ward level.

**Figure 1:**
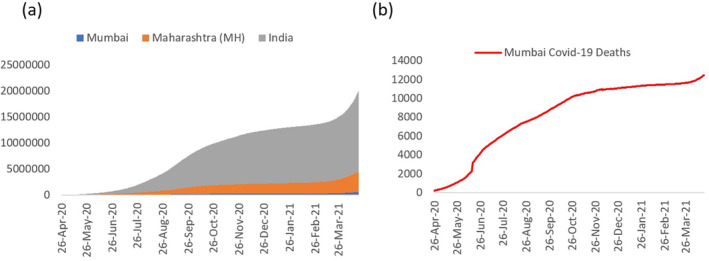
(a) Daily COVID‐19 cases in India, Maharashtra (MH), and Mumbai. (b) Total number of death due to COVID‐19 in Mumbai up till April 20, 2021. Note: COVID‐19 data for Mumbai is available from April 26, 2020 onwards. Source: https://api.covid19india.org/documentation/csv/.

**Figure 2:**
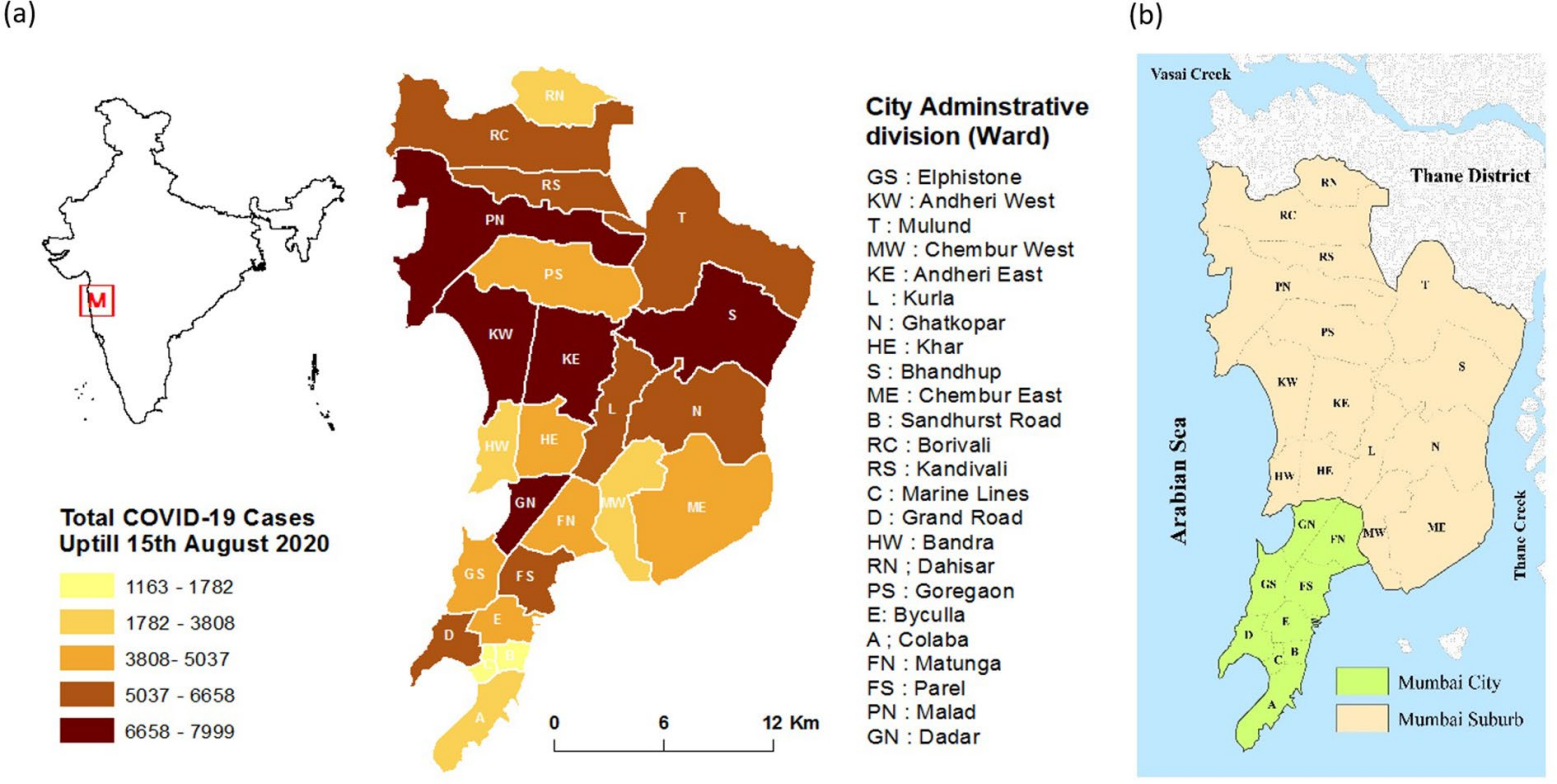
(a) Study area and ward wise breakdown of positive cases in Mumbai as of August 15, 2020. (b) Mumbai City and Mumbai Suburban limits. Sources: Prepared by authors, based on Brihanmumbai Municipal Corporation COVID‐19 Response War Room Dashboard.

India, located in the subtropical region, has been daily reporting its highest single‐day death toll due to COVID‐19 (Slater & Masih, 2020). The number of such cases is observed to be highest in the state of Maharashtra where Mumbai is the state capital and the most important financial hub of India. Figures 1 and 2 reveal the number of infected cases in India, Maharashtra, and Mumbai respectively. Mumbai is located on the western coast of India, along the Arabian Sea. During the mid‐18th century, seven islands of Bombay (currently Mumbai) were coalesced into a single landmass by bridging the islands (Murphy, 2013; Riding, 2018). Over time, Mumbai metropolitan area has been expanded further northwards through reclamation and over time got overcrowded due to proliferation of industries and services. The city is broadly classified into two zones‐the old city is located in the southern part and termed as “city” while the northern section is called the “suburban.” For ease of explanation, we divided Mumbai into southern zone (city or southern wards), and northern zone or suburbs, comprising of eastern, central and western wards/suburbs.

WHO's global air pollution database has ranked Mumbai as the fourth most polluted megacity in the world during 2010–2016 (WHO, 2018). System of Air Quality and Weather Forecasting (SAFAR) in India categorizes Air Quality Index (AQI) for pollutants into good, satisfactory, moderate, poor, very poor, and severe as 0–50; 51–100; 101–200; 201–300; 301–400; and 401 and above, respectively. In 2019, AQI in Mumbai ranged between 300 to 400—a range considered as very poor and unhealthy (Shinde, 2020). The city recorded 6% of total days with very poor AQI in 2019 as compared to 1%–5% in 2017–2018 (Chatterjee, 2019). During lockdown, particulate matter (PM) reduced and oxides of nitrogen and sulfur sharply declined, helping the city population to breath better air. Researchers pointed out that the improvement in air quality during the lockdown in 2020 restricted the transmission of the infection in some places due to reduction of pollutants (Manoj et al., 2020; Shehzad et al., 2020).

Mumbai slums were being identified as the biggest COVID‐19 clusters during the initial phases of 2020 lockdown. On April 14, 2020, 31% of Mumbai's containment zones were in slums; and by late June, it was closer to 96% (Venkatachalam & Memon, 2020). Over 50,000 new cases in September were observed and the upsurge in cases was found to be driven by infection spread among non‐slum areas (Banaji, 2020). Mumbai alone shares maximum burden of this infection among all districts in the country. COVID‐19 hotspots identified by the Municipal Corporation of Greater Mumbai (MCGM) were 750 in number in July 2020 and it sealed 5,831 buildings as on June 30, 2020. The doubling rate of cases in Mumbai worsened from 90 days recorded a week earlier to 49 days, as reported by the municipal corporation in March 2021 (Mint, 2021). Thus, the question arise as to why does this metro with a clear edge in economics and infrastructure and holding almost half the population of Spain, faces challenges to control the spread of coronavirus? This particular issue becomes an addressable concern when new findings suggest that improved air quality could help us in overcoming the pandemic. Based on existing literature, the paper answers the following questions: Does pollution exacerbates COVID‐19 infection in Mumbai? If so, which are the hotspots? Does pollutants act as determinants of COVID‐19 deaths when other factors are controlled? It is hypothesized that Mumbai shows substantial link between air pollution and COVID‐19 infection along with death rate after controlling other important city level factors, as far as the data allows.

## 2 Materials and Methods

Mumbai's recent ward wise data on COVID‐19 helped us to look into the aspect of spatial relation of disease spread and pollution. Among various air pollutants, the present study emphasizes on factors including concentration of SO_2_, NO_2_, and PM_10_ level that are proven to be accountable for triggering major cardiovascular‐respiratory diseases (Hosamane & Desai, 2013; Kumar et al., 2016; Maji et al., 2017; Shehzad et al., 2020). The unit of analysis is Mumbai ward that is, an administrative division of a city that typically elects and is represented by councilor. Mumbai is divided into 24 wards. Ward wise COVID‐19 data till August 15, 2020 was retrieved from the Brihanmumbai Municipal Corporation (BMC), Department of Health Portal (https://stopcoronavirus.mcgm.gov.in/).

To access the impact of air pollution across Mumbai, annual average concentration of SO_2_, NO_2_ and PM_10_ of past 3 years was calculated based on the Central Pollution Control Board (CPCB) data for the period of 2017–2019 (https://app.cpcbccr.com/AQI_India/). CPCB functions under the aegis of the Ministry of Environment, Forest, and Climate Change (MoEF & CC), Government of India and provides most authentic, real time, government approved environment related data for India. CPCB executes a nationwide program of ambient air quality monitoring, known as National Air Quality Monitoring Program (NAMP). The monitoring of pollutants is carried out for 24 h (4‐hourly sampling for gaseous pollutants and 8‐hourly sampling for particulate matter) with a frequency of twice a week, leading to one hundred and four (104) observations in a year (CPCB, 2010). CPCB estimates the Ambient Air Quality Measures as per Prevention and Control of Pollution, Act No.14 of 1981 in India. The details of Indian standard and WHO acceptable limits of the pollutants is shown in Table 1.

**Table 1:** National Ambient Air Quality Standard and WHO Acceptable Limits of the Pollutants With Standard Procedures of Measurement

Sl. No.	Pollutant	Time weighted average	Concentration in ambient air		WHO specification	Methods of measurement in India
			Industrial, residential, rural and other areas	Ecologically sensitive area (notified by central government)		
1	Sulfur dioxide (SO_2_) μg/m^3^	Annual	50	20	20 μg/m^3^ 24 h mean	Improved West and Gaeke Method
		24 h	80	80		Ultraviolet fluorescence
2	Nitrogen dioxide (NO_2_) μg/m^3^	Annual	40	30	40 μg/m^3^ annual mean	Modified Jacobs and Hochheiser Method (sodium arsenite)
		24 h	80	80		Chemiluminescence
3	Particulate matter (size less the 10 μg) or PM_10_ μg/m^3^	Annual	60	60	20 μg/m^3^ annual mean	Gravimetric
		24 h	100	100		TOEM (Tapered Element Oscillating Microbalance)
						Beta attenuation

*Note*. Compiled by the authors.

Source: https://cpcb.nic.in/uploads/National_Ambient_Air_Quality_Standards.pdf and air quality guidelines for particulate matter, ozone, nitrogen dioxide and sulphur dioxide. Global update 2005. Summary of Risk Assessment (WHO, 2006).

There are 10 pollution monitoring stations in Mumbai (CPCB, 2010). These monitoring stations are operated continuously by municipal corporation of Mumbai, National Environmental Engineering Research Institute (NEERI), and the State Pollution Control Board. We randomly checked the given data obtained from the monitoring station with the IQAir that provides the world's largest real time database of global air quality information through aggregating air quality data from a range of sources (https://www.iqair.com/world-air-quality). Both the data information reveal significant correspondence. To understand the spatial variability of the pollutant's concentration over the study area, we used ordinary Kriging interpolation techniques. Ordinary Kriging is a popular method used in health science, geochemistry, and pollution modeling to estimate the local spatial variations of the air quality data (Griffith, 2012; Jha et al., 2011; Kumar et al., 2016; Wong et al., 2004). According to Kumar et al. (2016), Kriging gives the best result for Mumbai among all other interpolation techniques like Inverse Distance Weighted (IDW), Gaussian decays, Spline, etc., as it uses a limited set of sampled data points to estimate the value of a variable over a continuous spatial field. Further, if there is at least moderate spatial autocorrelation, Kriging helps to preserve spatial variability that would be lost using a simpler method (Auchincloss et al., 2007). Additionally, GIS Zonal Statistics Mean Tool was used to estimate pollutants for each ward (Soysal et al., 2012).

To measure the 2020 ward wise population of Mumbai, WorldPop data set was used. This high resolution data set provides excellent information on the world's human population, allowing estimation of local population distribution and thus is a rich resource for spatial demographic analysis. The population data set is available at a resolution of 30 arc (approximately 1 km at the equator), adjusted and matched with the corresponding official data of the United Nations Population Division (UNPD) of the Department of Economic and Social Affairs of the United Nations (WorldPop, 2020). Ward wise population was calculated using GIS Zonal Statistics Sum for the cells falling within the ward‐polygons (Silva‐Coira et al., 2020; Soysal et al., 2012).

To estimate the possible air pollution effect on COVID‐19 cases in Mumbai, the health impact in terms of total infected population and assessment of the number of deaths were carried out across 24 wards. We considered the baseline annual averages of pollution concentration of SO_2_, NO_2_, and PM_10_ as 2, 2, and 41 μg/m^3^ respectively, for Mumbai (A. Joseph et al., 2003; Kumar et al., 2016; Shah & Nagpal, 1997). Health impact assessment was carried out using ward population, concentration data, and Concentration Response (CR) coefficients. CR functions are well accredited in supporting epidemiological evidence in calculating health impact assessments (Ren et al., 2017; Sheehan et al., 2016). It estimates the health risks attributed to any pollutant based on estimated epidemiological associations. The coefficient represents the percentage change in morbidity rate per mg/m^3^ change in pollution concentration, after controlling for other socio‐demographic factors (Patankar & Trivedi, 2011). In other terms, the CR coefficient represents the concentration corresponding to number of health risk cases. Average CR coefficient of each air pollutant in Mumbai, calculated by Kumar et al. (2016) and Patankar and Trivedi (2011) is given in Table 2. Health impact assessment was carried out using the following:

(1) $$\Delta H_{jp}=b_{p}\times {\text{POP}}_{j}\times \Delta {\text{AQ}}_{p}$$

ΔH_*jp*_: Change in health impact. Where, *j* represents a specific Mumbai ward and *p* represents the type of pollutant.
b_*p*_: Mean CR coefficient related to pollutant *p*.
POP_*j*_: Population of the particular ward of the city *j*.
ΔAQ_*p*_: Rate of change in the concentration of pollutant *p*.

**Table 2:** Concentration Response Coefficients of Pollutants for Health Impact

Respiratory infections	Concentration response (CR) coefficient %		
	SO_2_	NO_2_	PM_10_
Cough	–	0.021	0.007
Breathlessness	–	0.028	0.009
Wheezing	–	0.02	0.006
Cold	–	0.018	0.006
Cardiac ailments	0.118	–	–
Other chest illness	0.162	–	–
Allergic rhinitis	–	0.046	0.014
COPD	–	0.023	0.014
**Mean**	**0.140**	**0.026**	**0.009**

*Note*. CR coefficients are controlled for age, gender, smoking habit, distance traveled to place of work, occupation, width of road adjacent to the residence, presence of polluting industry near residence, hours spent in kitchen, quality of kitchen, ventilation and type of cooking fuel used.

Source: Adopted from Kumar et al. (2016) and Patankar and Trivedi (2011).

To summarize the method, first the ward wise COVID‐19 total cases was computed till August 15, 2020. Next, for estimating the long‐term impact of air pollutants, 3‐year annual average (2017–2019) concentration was calculated and represented in a thematic map. To estimate the population suffering from health problems due to pollution exposure, current population was adjusted for each pollutant. The CR estimates used in the study were controlled for other socio‐demographic factors.

To check the association of air population and the COVID‐19 positive cases, we applied bivariate local indicator of spatial autocorrelation (LISA). Additionally, to measure local variation between two matrices, bivariate Moran's *I* statisti*c* was applied with a randomization test on a Z‐score with 999 permutations as follows:

(2) $$\text{Bivariate}\;\text{Moran}’s\;I=\frac{n}{S_{0}}\times \frac{\sum_{i}\sum_{j}W_{ij}\left(x_{i}-\bar{X}\right)\left(y_{j}-\bar{Y}\right)}{\sum i{\left(y_{i}-\bar{Y}\right)}^{2}}$$

x: Independent variable, total COVID‐19 cases in Mumbai
y: Dependent variable, SO_2_, NO_2_, and PM_10_ of Mumbai
$\bar{X}$: Mean of *x*
$\bar{Y}$: Mean of *y*
$W_{ij}$: Standardized weight matrix between observation *i* and *j* with zeroes on the diagonal
$S_{0}$: Aggregate of all spatial weights, that is, *S*_0_ = *P*_*i*_ *P*_*j*_ *×* *W*_*ij*_.

Furthermore, to examine the association between COVID‐19 death rate and pollution at ward level, we regressed a set of ecological, demographic and infrastructural variables like population density, slum concentration, health facility, road density, *nullah* or drainage, and police station. There are data constraints while doing this analysis, for example, we do not have detailed data of individual COVID‐19 cases; comprehensive climate data are not available at ward level as the municipality provides weather related information only at city level. So we restricted our analysis within the frame of data availability provided by authentic government sources. COVID‐19 death and infection, being highly correlated, we regressed death rate as it provided better *R* ^2^ values as compared to the regressions on infected cases. Through Ordinary Least Square model (OLS) estimation, we checked the relation of COVID‐19 death rates with population exposed to ill health due to various pollutants, controlling other ecological determinants. Spatially adjusted regression models were used further for correcting the spatial endogeneity bias. As we know that nearby things are similar, and ordinary least squares considers individual cases as independent, we applied spatial lag model (SLM) and spatial error model (SEM). SLM relates a set of independent variables on an outcome variable that are auto regressed on spatially lagged response variables; while SEM accounts for the spatial dependency by an error term (Khan et al., 2018; Shaw et al., 2020).

## 3 Results

Table 3 reveals summary statistics of Mumbai on COVID‐19 infection, population density, population exposed to specific pollutants, and city infrastructure. Mean population exposed to SO_2_, NO_2_, and PM_10_ as estimated by authors were 415 thousand, 238 thousand and 0.78 thousand respectively. Estimated ward population density was 33 thousand per sq. km. The average number of slums per ward in Mumbai was 100, mean number of drain was 31, average health facility and police station per ward were four each, and road density was around 6 km/sq. km. It evidently reveals the high population concentration and overloaded infrastructure in the city.

**Table 3:** Description of Model Variables in Wards and Summary Sample Statistics of Mumbai, August 2020

Variables at ward level	Data source and description	Mean	Std. Dev.
Population density/sq. km	Population density is measured by the authors using World Population Project	33,362.85	8,533.51
COVID‐19 positive cases	COVID‐19 Response War Room Dashboard (BMC)	5,142.96	1,774.37
COVID‐19 death rate	Ward wise number of deceased due to COVID‐19 per million population (MCGM)	62.30	33.16
Population exposed to SO_2_	Exposed population by specific pollutants has been estimated by the author using pollution concentration data from CBCS (2017–2019) (CPCB)	415,109.90	272,736.20
Population exposed to NO_2_		237,991.10	129,858.90
Population exposed to PM_10_		7,853.41	3,118.63
Number of slums	Ward wise directory information about various infrastructural development (MCGM)	100	70.85
Number of drains/*nullahs*		31.71	32.23
Number of health facility		4.17	2.24
Density of roads in km/sq. km		5.62	4.92
Number of police stations		4.17	1.35

*Note*. Slums are defined as per Census 2011; health facility is defined as the total number of public and private hospitals; density of the roads is the length of the roads per area of the ward.

Data sources: https://stopcoronavirus.mcgm.gov.in/; http://dm.mcgm.gov.in/ward-directory; https://portal.mcgm.gov.in/irj/portal/anonymous and https://www.worldpop.org/project/categories?id=3.

### 3.1 Extent of Average Air Pollution in Mumbai

**Figure 3:**
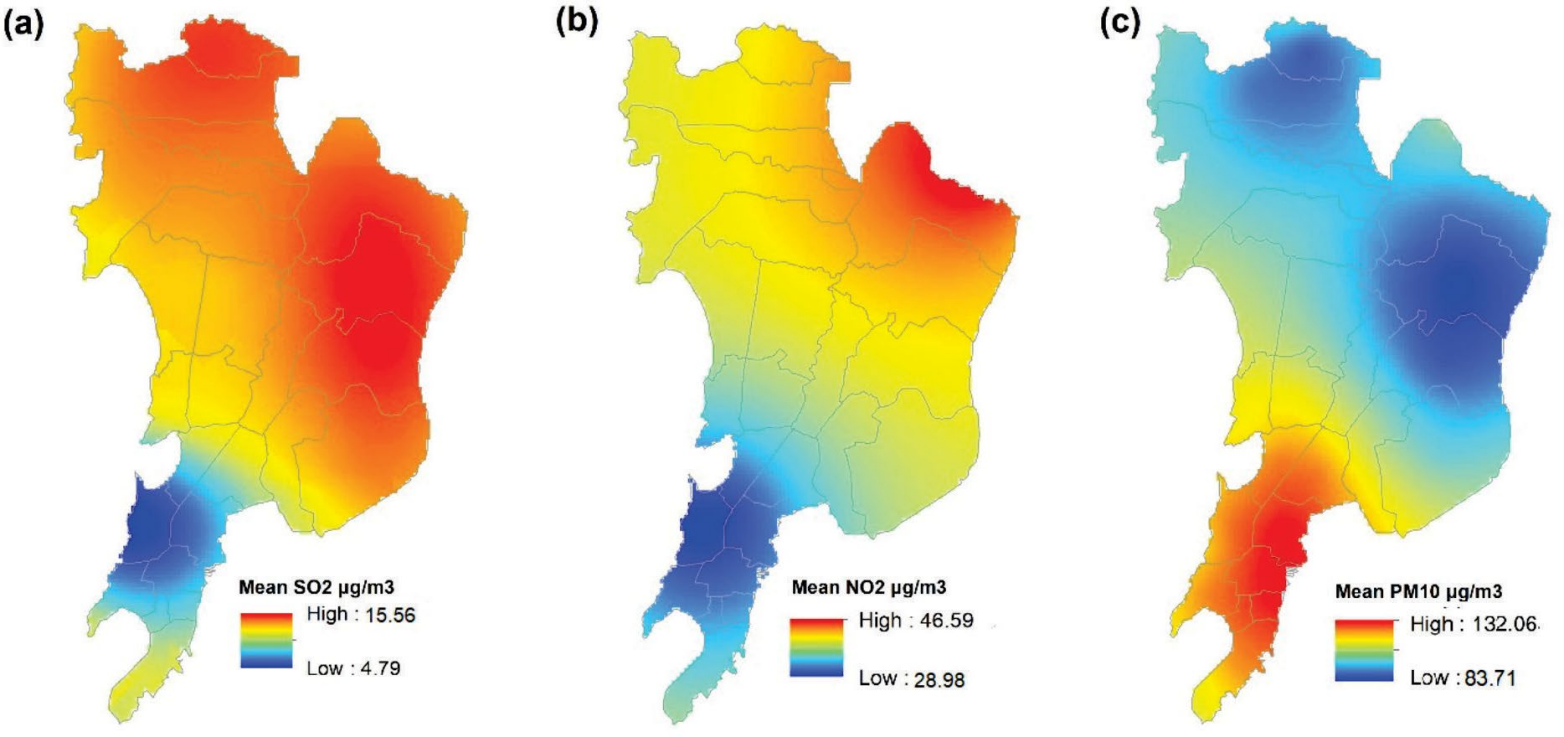
Three years’ annual average concentration of air pollutants (a) SO_2_, (b) NO_2_, and (c) PM_10_ across Mumbai (2017–2019). Source: prepared by authors.

Annual average concentration (2017–2019) of air pollutants that is, SO_2_, NO_2_, and PM_10_ across different wards of Mumbai is observed in Figure 3. The long‐term annual mean of NO_2_ was 40 μg/m^3^ and SO_2_ was 20 μg/m^3^ (WHO, 2006). Concentration of NO_2_ and SO_2_ was higher in the suburban zone of the study area (Figures 3b and 3a). NO_2_ close to 50 μg/m^3^ was prominently observed in the north eastern fringe of Mumbai. The annual average of SO_2_ concentration was highest across the wards of eastern and northern suburbs, that is, S (15.55 μg/m^3^), followed by the adjoining wards of N (15.23 μg/m^3^) and T (15.04 μg/m^3^). The least concentration of SO_2_ was in the southern coastal wards of GS (5.01 μg/m^3^), FS (6.87 μg/m^3^) and E (6.88 μg/m^3^). Similarly, NO_2_ was recorded highest across the eastern wards that is, T (38.57 μg/m^3^), S (38.24 μg/m^3^) and N (37.51 μg/m^3^); while the least concentration of this pollutant was detected in the wards of GS (29.75 μg/m^3^), E (29.95 μg/m^3^) and D (30.09 μg/m^3^) (Figure 3b).

PM_10_ was found to be higher than the WHO standard across all the wards in Mumbai. The long‐term permissible mean value of PM_10_ was 20 μg/m^3^ per year, while short‐term mean value was 50 μg/m^3^ for 24‐h. The southern wards namely, FS (131.93 μg/m^3^), E (130.85 μg/m^3^), and B (130.63 μg/m^3^) recorded higher concentration of PM_10_ that might be due to the presence of marine aerosols. While the wards in the western suburb of Mumbai, that is, S (83.45 μg/m^3^), N (83.45 μg/m^3^) and RN (88.83 μg/m^3^) showed a comparatively lower concentration (Figure 3c) of PM_10_. The standard deviation of SO_2_, NO_2_, and PM_10_ are 3.10 μg/m^3^, 2.99 μg/m^3^, and 15.73 μg/m^3^ respectively.

### 3.2 Population Exposed to Health Risks Due to Air Pollution in Mumbai

**Figure 4:**
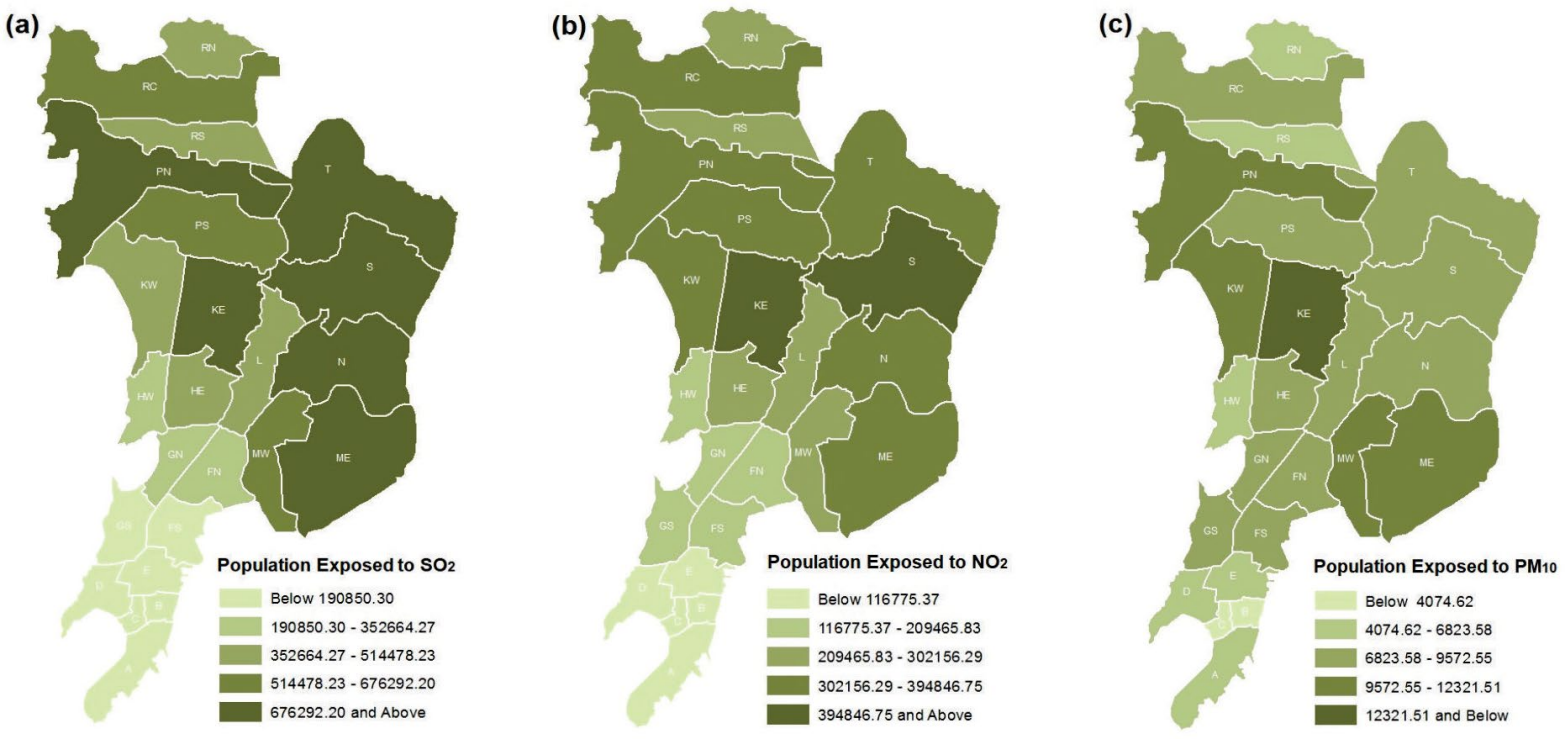
Population with health morbidity due to exposure to (a) SO_2_, (b) NO_2_, and (c) PM_10_. Source: prepared by authors.

The population exposed to long‐term air pollutant with health risk was calculated using the UN adjusted 2020 WorldPop data set (Table S1a). The maximum population exposed to health risk due to SO_2_ was in KE (838,106) ward followed by S (837,138) and N (784,706) wards. The least exposure of population to SO_2_ were observed in B, C, and GS wards (Figure 4a). While maximum bearing on population for exposure to NO_2_ was evident in the northern wards that is, KE (487,537), followed by S (415,717) and N (391,096); the lowest exposure was visible in the southern wards namely, B (24,085), C (31,313), and D (87,232) (Figure 4b). Although the concentration of PM_10_ was much higher than the permissible limit in Mumbai (WHO, 2006), the impact of PM_10_ was comparatively lower than exposure to NO_2._ The mean CR coefficient of various respiratory morbidity due to PM_10_ was only 0.009. It was observed that the maximum CR coefficient of chest illness (0.162) was due to exposure to SO_2_ followed by allergic rhinitis (0.046) caused by NO_2_ (Table 2). The maximum impact of PM_10_ was observed in the suburban zone, that is, KE ward (15,070) followed by ME (12,006) and PN (11,143). On the contrary, population with the least risk of exposure was in the southern wards of B (1,326), C (1,673), and D (4,555). A significant positive Pearson's correlation coefficient between COVID‐19 cases and population exposed to SO_2_ (0.5668), NO_2_ (0.6405), and PM_10_ (0.6691) with *p*‐value <0.05 indicated that pollution related morbidity prompts the COVID‐19 infection across Mumbai.

### 3.3 Spatial Autocorrelation of Air‐Pollution and COVID‐19 Infection

**Figure 5:**
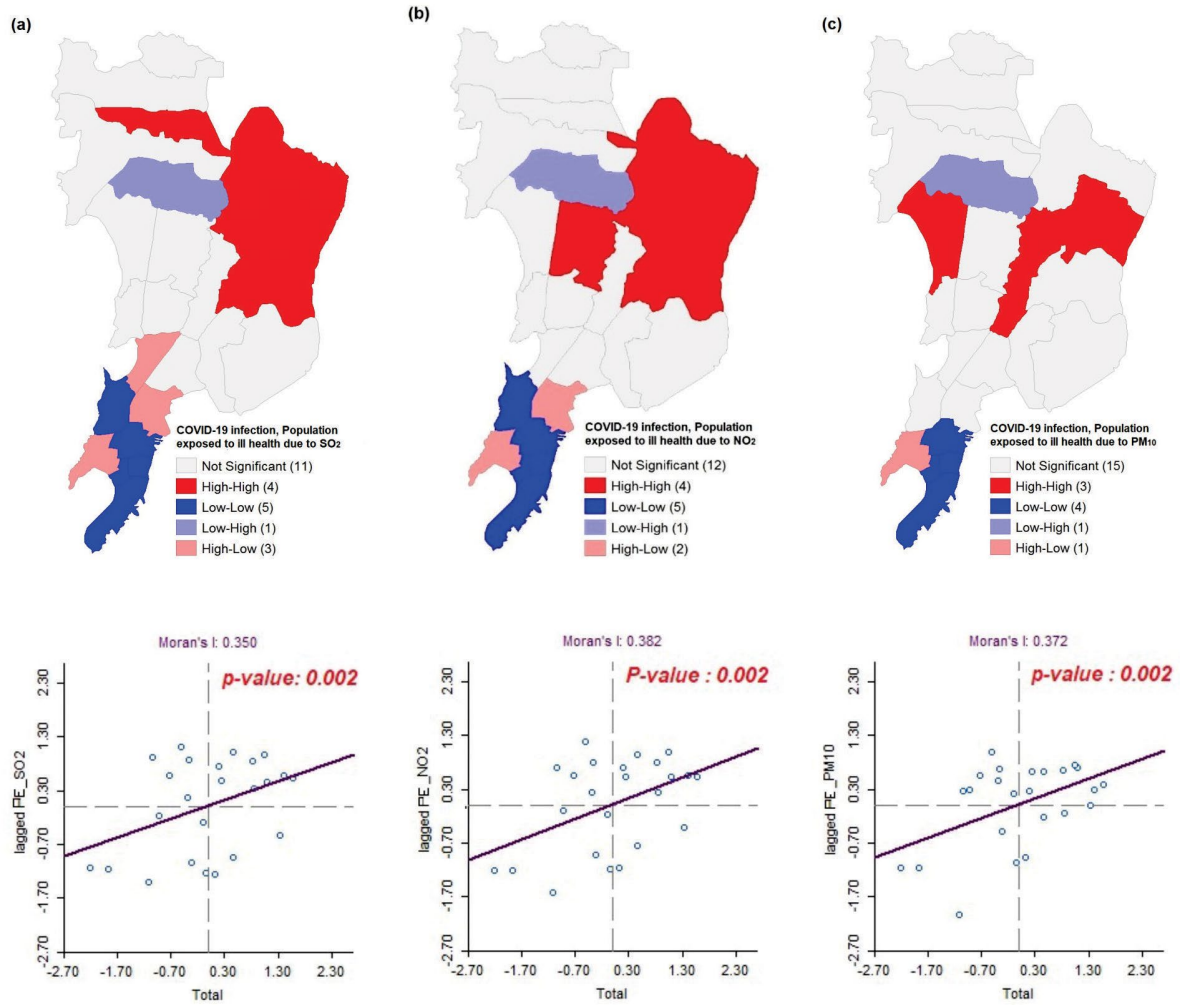
Bivariate LISA cluster maps showing the spatial clustering of total COVID‐19 cases with population exposed to ill health due to (a) SO_2_, (b) NO_2_, and (c) PM_10_ across Mumbai up till August 15, 2020.

To access the heterogeneity of spatial clustering of COVID‐19 infection and air pollution, bivariate LISA and Moran's *I* statistics was constructed (Figure 5). LISA maps show compelling evidence of association between COVID‐19 cases and ill health arising out of exposure to pollution in Mumbai. The ward wise scenario can be visualized as:

- *Hot Spot:* (red) high‐ill health from exposure to pollution and high COVID‐19 infection
- *Cold Spot:* (light blue) low‐ill health from exposure to pollution and low COVID‐19 infection
- *Spatial Outliers*: (light blue) low‐ill health from exposure to pollution and high COVID‐19 infection
- *Spatial Outliers*: (light red) high‐ill health from exposure to pollution and low COVID‐19 infection

The high‐high spatial clustering was found across four northern wards namely, S, N, T, and PN where higher population suffering from ill heath due to SO_2_ corresponded with high COVID‐19 cases. Whereas low‐low clustering was visible across five southern wards namely, GN, E, C, B, and A (Figure 5a) where spatial lagged Moran's *I* was 0.350 at 1% significance level. Similarly, the high‐high clustering of population suffering from ill health due to NO_2_ and COVID‐19 was detected across four wards of the northern suburbs—T, S, N, and KE while, low‐low clusters were found in five wards of the city, that is, GN, E, C, B, and A (Figure 5b). The bivariate lagged Moran's *I* was 0.382 with *p‐value* 0.002. High‐high clustering of COVID‐19 infected people and population suffering from ill health due to PM_10_ was found in the low lying wards of suburban Mumbai. KW, S, and L ward experienced the maximum autocorrelation in this regard. While wards in the southern part had the low‐low clustering with Moran's *I* = 0.372 where the *p*‐value is 0.002. Thus, suburban areas of Mumbai (stretching from Vikroli, Mulund, Bhandup, Ghatkopar, Kurla to Andheri, Joyeswari, Vile Parle, Malad) revealed high association between different pollutants and COVID‐19 cases. This signifies that substantial population exposure to pollutants in these areas were highly associated with COVID‐19 infection (Figure 5c).

### 3.4 COVID‐19 Death and Pollution: OLS and Spatial Regression Analysis

Table 4 shows the long‐term exposure to various pollutants exacerbating COVID‐19 related deaths across wards of Mumbai. We considered number of deceased in this section due to better predictability of the models. In all the three models shown in Table 4, NO_2_ exposure comes out to be a statistically significant predictor of COVID‐19 death. OLS model estimation shows that population exposed to ill health due to NO_2_ (*β* = 0.001017, *p*‐value <0.01) and number of waste water drains (*β* = 0.39523) were positively associated with COVID‐19 death rates at 5% level of significance. It means, with unit increase in population exposure to NO_2_ and number of drains, there is significant increase in COVID‐19 deaths in Mumbai.

**Table 4:** Multivariate Spatial Regression Models Showing the Effect of City Level Variables on COVID‐19 Death

Predictors	Coefficient (std. error)		
	OLS	SLM	SEM
Population exposed to ill health due to SO_2_	−0.00045(0.00014)	−0.00046***(0.00011)	−0.00036***(0.00008)
Population exposed to ill health due to NO_2_	0.00101***(0.00038)	0.00102***(0.00029)	0.00079***(0.00024)
Population exposed to ill health due to PM_10_	−0.00976*(0.00460)	−0.00974***(0.00349)	−0.00876***(0.00276)
Population density/sq.km	0.00116(0.00075)	0.00119**(0.00057)	0.00107**(0.00051)
Number of slums	−1.34161(2.62655)	−1.66959(2.03174)	−1.35085(2.38768)
Number of drains/*Nullahs*	0.39523**(0.16397)	0.37738**(0.12461)	0.22719**(0.11502)
Number of public health facility	−0.00615(0.81717)	0.11782(0.62994)	0.63261(0.71667)
Density of roads/sq.km	2.11824(1.0737)	2.04531**(0.81342)	0.94990(0.81112)
Number of police stations	3.74319(2.62076)	4.10434**(2.04953)	3.39332(2.74343)
Constant	6.98352(30.9843)	14.5009(24.8804)	26.9782(16.1254)
*ρ*		−0.116,778	
*λ*			−0.910,806
AIC	212.998	214.645	208.737
*R* ^2^	0.827,322	0.830,436	0.882,551

Abbreviations: OLS, Ordinary Least Square Model; SLM, Spatial Lag Model; SEM, Spatial Error Model.

Standard errors in parentheses, ***p < 0.01, **p < 0.05.

SLM estimation illustrates that with increase in population density (*β* = 0.00119), number of drains (*β* = 0.37738), road density (*β* = 2.04531) and number of police stations (*β* = 4.10434), COVID‐19 death rate increased significantly. Like OLS, population suffering from ill health due to NO_2_ was highly and positively prone to COVID‐19 fatality (*β* = 0.00102) in SLM. Interestingly, while adjusting the spatial autocorrelation, both density of roads and number of polices stations lost the significance in SEM. The findings also signify that populous wards of Mumbai in terms of population density and drains suffered higher COVID‐19 casualty. Under spatial models, SEM gives a better fit with lowest AIC value (208.737). The highest R^2^ was estimated for SEM (0.882551) followed by SLM and OLS (0.830436; 0.827322) respectively. However, population exposed to SO_2_ and PM_10_ reveal a negative association with COVID‐19 death in SLM and SEM at 1% level of significance.

## 4 Discussion

The long‐term air pollution exposure to particulate matters, NO_2_ and SO_2_ leads to respiratory inflammation and series of respiratory complications. Literature suggests that air pollution has considerably worsened the COVID‐19 outbreak and spread in Europe and in the USA due to the reason that in areas where people lived with polluted air for decades, that very polluted air acted as vehicle for viral transmission (Bashir et al., 2020; Berman & Ebisu, 2020; Fattorini & Regoli, 2020; Wu et al., 2020). In India, cities are more adversely affected by COVID‐19 as compared to its rural parts. Mumbai is the worst‐hit in this regard. In spite of a definite edge in terms of facilities, the hurdle faced by Mumbai in managing COVID‐19 is a matter of deep concern. It is therefore imperative to understand the role of air pollution in this context of COVID‐19 spread and associated fatality, as Mumbai usually experiences poor to very poor and unhealthy air quality (Borwankar, 2020).

The paper, first of its kind in India, addresses the question whether pollution is accentuating COVID‐19 prevalence in Mumbai and if so, which are the spatially significant hotspots. The paper also explains whether pollutants act as determinants of COVID‐19 death when other factors like population density, health infrastructure, number of slums, waste water drains and roads are controlled. The study investigates the relationship between population exposed to specific pollutants that is, SO_2_, NO_2_, PM_10_, and COVID‐19 infection and deaths in administrative wards (smallest administrative unit of urban places in India) of Mumbai using government data through spatial autocorrelation and different regression models.

The study reveals that annual average (2017–2019) of PM_10_ is much higher than the WHO standard across all the wards of Mumbai. Overall, the population in the suburban part of Mumbai are at a higher risk of respiratory morbidity as pollutants like NO_2_ and SO_2_ are reported to be more in the suburbs. Population exposure to air pollutants leads to more respiratory morbidity and such exposure is highly associated with total COVID‐19 cases, as observed in the study. Significant positive link with infected population and population exposed to SO_2_ (0.5668), NO_2_ (0.6405), and PM_10_ (0.6691) supports the hypothesis that pollution and COVID‐19 infection are very much associated in Mumbai metropolis. High exposure to specific pollutants and high‐presence of COVID‐19 cases are well established in certain wards in the suburban parts of the city while southern wards show cold‐spots, that is, low pollution exposure and low infected population. The study further demonstrates that when other variables are controlled, factors like NO_2_ concentration, population density and presence of waste water drains have significant positive relationship with COVID‐19 death rate in Mumbai. However, negative relation of SO_2_ and PM_10_ with the infection demands further research. A hand full of studies across Asia already established a negative or insignificant association of COVID‐19 and PM_10_. For example, the study conducted across 3‐cities of China by Jiang et al. (2020) revealed a negative relationship of PM_10_ with COVID‐19 (*β* = −0.037; *β* = −0.04; and *β* = −0.089). Similarly, Gupta et al. (2021) revealed a negative association of COVID‐19 lethality with PM_10_ (*β* = −4.56, *p*‐value = 0.281) in different cities of India, Pakistan, Indonesia, and China. Furthermore, SO_2_ is associated with a 7.79% decrease (95% CI: −14.57 to −1.01) in COVID‐19 confirmed cases of Wuhan City, China (Zhu et al., 2020). Studies indicate that particulate matter at ground level during cold and dry winter and during spring seasons usually have high adverse impact on COVID‐19 infection spread (Zoran et al., 2020). Mumbai, being in the subtropics with high temperature and humidity, perhaps has not experienced the ill effect of PM_10_ in context of COVID‐19.

The long‐term air pollution exposure leads to respiratory inflammation, coughing, bronchitis, asthma attacks etc (WHO, 2000). Pollution levels depend upon a number of factors, like topography, building density, road density, weather conditions, solid fuel use etc. Pollutions often build up in low lying areas that is, valleys, between hills etc. Mumbai, a primate city, has a unique geophysical location. It is on the Arabian sea coast, with large reclamation land and bounded by the hills on the eastern border. Rapid economic growth in Mumbai had led to a substantial increase in the level of SO_2_, No_2_, and PM_10_ over time (Hosamane & Desai, 2013; Maji et al., 2017). In addition, research has identified that in Mumbai, prevalence of diminished lung function, acute and chronic respiratory symptoms such as cough and wheeze, asthma has increased in areas with elevated levels of air pollution (Gordon et al., 2018). A study in Mumbai concludes that the slum areas bear the exposure burden (expressed as a product of population and daily exposure) to as high as 80% in the city (Srivastava & Kumar, 2002). Few stimulating researches published recently by Fattorini and Regoli (2020), Ogen (2020), and Zhu et al. (2020) expressed that 78% of deaths due to COVID‐19 occurred in just five regions in northern Italy and Spain that have the highest concentrations of NO_2_. The papers based on Italy and Spain argues that Lombardy region in the Po Valley and Madrid administrative region which are ringed by mountains and which therefore experiences the downward air pressure have been the worst‐hit regions of these two countries. Besides pollution, recent study of Manoj et al. (2020) indicates possible triggering of COVID‐19 transmission and air pollution under moderate‐to‐high humidity conditions. Needless to mention, Mumbai receive heavy monsoon rain and is located in the tropical coast resulting in humid climatic condition for significant parts of the year.

In the above context, our findings can be well explained with existing research support as follows:

First, similar to many European cities, Mumbai is built up on sea through reclamations and has distinct low lying areas mainly in the suburban part (Figure S1a); the areas in our study that reveal hot spots for COVID‐19 and pollution exposure are closely matching with low lying areas or wet lands of Mumbai suburbs that faces maximum water logging during monsoon rains, that is, Kurla, Sion, Matunga, Mulund, Kalina, Ghatkopar, Juhu, Santacruz, and Andheri (Figure S1a). The monsoon of 2020 has deteriorated the situation further in Mumbai. As reported by some leading newspapers, in the initial phase when COVID‐19 cases were spotted in an area, the municipality workers would disinfect that neighborhood and seal off the affected area. But with the advent of heavy rains, such precautionary measures turned irregular in the city (P. J. Joseph, 2020). Mumbai drainage system is more than 100 years old and it is dominated by open drains. Further, the storm water drain network can drain out only 25–50 mm water per hour and Mumbai often receives heavy rainfall crossing 200 mm (Singh, 2020). In 2020, Mumbai and its surrounding witnessed heavy monsoon downpour, Cyclone *Nisarga* (first storm of its intensity post 1891) leading to waterlogging, poor sanitation, delayed treatment due to transport bottlenecks, rapid spread of other infections leading to overburdening of health facilities and delayed timely measures for controlling COVID‐19. Stimulating, our study finds that presence of drain significantly increased the chances of COVID‐19 deaths, which could be associated with sanitation related problems during monsoon in the city. Thought provoking research around the world are now pursuing to analyze sewerage water for COVID‐19 tracing with the hope that wastewater data can supplement as an additional attribute in explaining COVID‐19 prevalence. SARS‐CoV‐2, has already been detected in wastewater not only in the West but in India as well (Kaul, 2020; Larsen & Wigginton, 2020). Peccia et al. (2020) demonstrated that concentrations of SARS‐CoV‐2 RNA in primary sewage sludge was in conformity of being an evidence of the local spread of COVID‐19 cases and an explanatory variable for the recorded increase in hospital admissions in parts of the USA. Atmospheric loading of coronaviruses in water droplets from wastewater is yet to be understood but could provide a more direct respiratory route for human exposure, particularly near waterways that are receiving wastewater (Flockhart, 2020).

Second, the air flow during most of the months in Mumbai is mainly from the south west to the north east direction. Hills obstruct the air flow at the eastern border of the city leading to concentration of pollutants within the city boundary. Mumbai ranks fourth in the world ranking in traffic congestion, which clearly gives an idea of the source of pollution across the city (Traffic Index, 2019). The city ranks twentieth in the latest AQI of polluted city ranking in the world (IQAir, 2020). Of all types of emissions analyzed in the research, the maximum contribution of different pollutants is from the industry, followed by the transport system (Telang, 2018). Our study reveals that the low‐low association of pollutants with COVID‐19 in the southern wards and that could be due to less number of industries, high land price, predominance of corporate offices, defense areas etc., and partly for its geographic position‐that is, having open sea in both the western and eastern border, due to which wind is free flowing. Although association of the novel virus and pollution are still to be established, chronic exposure to atmospheric pollution and compromised respiratory system may stand as a risk factor in influencing the spread and fatality of COVID‐19. Our study, suggests strong relation of this viral infection with pollutants in Mumbai, especially in the suburbs which are more populated and more polluted as compared to the southern wards.

Third, association of NO_2_ and COVID‐19 are well explained in different studies, though contrasting results exist. Ran et al. (2020) found no clear effects of NO_2_, SO_2_, and CO on the initial transmissibility of COVID‐19 across Chinese cities. While findings of Copat et al. (2020) highlight the important contribution of PM_2.5_ and NO_2_ as triggering of the COVID‐19 spread and its lethality. Researchers like Bashir et al. (2020), Fattorini and Regoli (2020), Ogen (2020), and Zoran et al. (2020) proved significant association with COVID‐19 and NO_2_ as is observed in our study. NO_2_ being the primarily emission from transportation and fuel combustion, has become an environmental pollutant in deteriorating air quality (Grange et al., 2019; Maawa et al., 2020). Based on scientific evidences, interesting observation by Ramachandran et al. (2013), reveals that the Mumbai and Pune (a neighboring city of Mumbai) region are well known emission hot spots for NO_2_ and spread of NO_2_ up to a few kilometers over the Arabian Sea in the Mumbai coast is well observed. In Mumbai region NO_2_ emissions come from industrial sector and other fugitive emissions (Table S1b). Mumbai's air has seen an increased amount of nitrogen dioxide (NO_2_) pollutants, in the recent past mainly due to increase in private vehicles in the city along with unchecked emission from petro‐chemical industries leading to chronic coughing, sore throat, among other respiratory problems (Telang, 2018).

Our findings, within all data limitations, therefore suggest that air quality is an important element to be addressed in disease management and sustainable urban development. However, the study has some limitations. First and foremost, we considered only Mumbai City with limited areal span and data points. This is mainly due to absence of detailed data at the smallest administrative urban level, in most of the urban areas of India. Mumbai municipality must be applauded for providing such a fantastic data set on COVID‐19. Second, we could not include some important determinants of COVID‐19 infection, such as age, gender, health behavior, co‐morbidity, testing rate, severity of cases, socio economic condition of individuals as well as some climatic variables like wind flow, temperature profile, planetary boundary layer height, etc., due to unavailability of data at micro level i.e., ward. Third, due to limited data points, we could not do more detailed or more sophisticated statistical analysis. We need more studies especially in Indian context to fill these gaps for more comprehensive understanding of environmental pollution impact in context of COVID‐19 spread in India by analyzing satellite data.

## 5 Conclusions

The world is at a critical juncture as COVID‐19 novel virus is extracting a huge toll on human life and economy. Due to dysregulated immune system caused by long‐term exposure to air pollution, people of Mumbai are probably more likely to suffer from COVID‐19. Distinct relation of pollution exposure and COVID‐19 infection as identified in this study, has perhaps worsened by level of pollution in this coastal city. The coronavirus crisis, however presents an opportunity for Mumbai in specific and the world as a whole to invest in cleaner fuel, more efficient public transport and more sustainable municipal infrastructure. Our empirical findings, first of its kind in India in context of this novel virus, call for implementation of ecology friendly policies in metropolis like Mumbai to save more lives. How seriously development should take environment into consideration in formulating integrated decision making is a fundamental question facing humanity. Association of COVID‐19 and environmental pollution should be considered as a measure of an integrated approach in sustainable development as it has strong implications for mitigation strategies related to the novel virus.

## Conflict of Interest

The authors declare no conflicts of interest relevant to this study.

## Supporting information

Supporting Information S1

## Data Availability

Mumbai ward wise COVID‐19 data is retrieved from BMC, Department of Health Portal https://stopcoronavirus.mcgm.gov.in/. The air pollutants concentration of SO_2_, NO_2_, and PM_10_ was collected from the Central Pollution Control Board (CPCB) for a period of 2017–2019 https://app.cpcbccr.com/AQI_India/.
