# Supplementary material for: Association Between Air Pollution and COVID‐19 Pandemic: An Investigation in Mumbai, India
Source: Geohealth. 2021 Jul 1;5(7):e2021GH000383. doi: 10.1029/2021GH000383 (PMC8287720; doi:10.1029/2021GH000383)
Supplement: Supplementary file 1 — Supporting Information S1 [file GH2-5-e2021GH000383-s001.docx]

Title: **Association between Air Pollution and COVID-19 Pandemic: An Investigation in Mumbai, India**

Aparajita Chattopadhyay^a^ and Subhojit Shaw^a^

^a^International Institute for Population Sciences, Mumbai

| **Mumbai Ward** | **Baseline SO_2_** | **SO_2_ (2017-19)** | **Rate of Change in SO_2_** | **Baseline NO_2_** | **NO_2_ (2017-19)** | **Rate of Change in NO_2_** | **Baseline PM_10_** | **PM_10_ (2017-19)** | **Rate of Change in PM_10_** | **Total Population** | **Population Exposed to SO_2_** | **Population**  **Exposed to NO_2_** | **Population**  **Exposed to PM_10_** |
| --- | --- | --- | --- | --- | --- | --- | --- | --- | --- | --- | --- | --- | --- |
| RN | 2 | 15.03 | 6.52 | 2 | 36.48 | 17.24 | 41 | 88.83 | 1.17 | 472510.45 | 431100.79 | 211808.32 | 5145.23 |
| RC | 2 | 14.77 | 6.38 | 2 | 36.18 | 17.09 | 41 | 92.13 | 1.25 | 743633.57 | 664629.42 | 330386.04 | 8654.66 |
| PN | 2 | 13.73 | 5.87 | 2 | 36.44 | 17.22 | 41 | 99.90 | 1.44 | 831062.94 | 682594.03 | 372043.98 | 11143.40 |
| HW | 2 | 12.30 | 5.15 | 2 | 33.59 | 15.80 | 41 | 116.48 | 1.84 | 343723.34 | 247839.72 | 141157.16 | 5906.12 |
| KW | 2 | 12.75 | 5.37 | 2 | 36.17 | 17.08 | 41 | 107.40 | 1.62 | 681990.34 | 513147.24 | 302931.74 | 10309.07 |
| T | 2 | 15.04 | 6.52 | 2 | 38.57 | 18.28 | 41 | 93.51 | 1.28 | 799942.85 | 730077.43 | 380285.76 | 9562.78 |
| RS | 2 | 14.25 | 6.13 | 2 | 36.97 | 17.49 | 41 | 94.94 | 1.32 | 487703.81 | 418364.83 | 221727.67 | 5988.67 |
| PS | 2 | 13.57 | 5.78 | 2 | 37.33 | 17.67 | 41 | 100.58 | 1.45 | 672877.62 | 544810.05 | 309047.79 | 9126.35 |
| L | 2 | 13.69 | 5.84 | 2 | 36.51 | 17.26 | 41 | 100.33 | 1.45 | 607086.71 | 496710.73 | 272366.70 | 8198.79 |
| KE | 2 | 13.10 | 5.55 | 2 | 36.77 | 17.39 | 41 | 102.38 | 1.50 | 1078480.55 | 838106.17 | 487537.21 | 15070.48 |
| S | 2 | 15.55 | 6.78 | 2 | 38.24 | 18.12 | 41 | 83.45 | 1.04 | 882433.60 | 837138.35 | 415717.29 | 8528.10 |
| N | 2 | 15.23 | 6.62 | 2 | 37.51 | 17.76 | 41 | 88.45 | 1.16 | 847097.40 | 784706.08 | 391095.67 | 9149.38 |
| HE | 2 | 12.50 | 5.25 | 2 | 34.73 | 16.37 | 41 | 114.92 | 1.80 | 538107.85 | 395671.38 | 228971.78 | 9054.44 |
| MW | 2 | 12.81 | 5.40 | 2 | 35.04 | 16.52 | 41 | 110.49 | 1.69 | 686463.58 | 519344.37 | 294881.37 | 10859.14 |
| ME | 2 | 14.05 | 6.02 | 2 | 36.25 | 17.13 | 41 | 101.48 | 1.48 | 871984.39 | 735362.33 | 388292.23 | 12006.30 |
| FN | 2 | 10.39 | 4.19 | 2 | 32.87 | 15.44 | 41 | 125.14 | 2.05 | 463604.32 | 272142.38 | 186060.40 | 8879.75 |
| FS | 2 | 6.87 | 2.44 | 2 | 30.56 | 14.28 | 41 | 131.93 | 2.22 | 395181.88 | 134806.84 | 146708.65 | 8179.94 |
| B | 2 | 8.38 | 3.19 | 2 | 30.51 | 14.26 | 41 | 130.63 | 2.19 | 64974.62 | 29036.34 | 24084.91 | 1325.66 |
| GN | 2 | 9.64 | 3.82 | 2 | 32.17 | 15.09 | 41 | 125.00 | 2.05 | 366597.71 | 196037.17 | 143800.21 | 7010.24 |
| GS | 2 | 5.01 | 1.51 | 2 | 29.75 | 13.87 | 41 | 126.27 | 2.08 | 365255.29 | 76978.19 | 131763.01 | 7090.09 |
| E | 2 | 6.88 | 2.44 | 2 | 29.95 | 13.98 | 41 | 130.85 | 2.19 | 280107.09 | 95627.51 | 101779.83 | 5729.28 |
| C | 2 | 8.64 | 3.32 | 2 | 30.47 | 14.23 | 41 | 127.88 | 2.12 | 84611.66 | 39339.27 | 31313.43 | 1673.33 |
| A | 2 | 11.08 | 4.54 | 2 | 31.86 | 14.93 | 41 | 123.12 | 2.00 | 285381.16 | 181488.04 | 110794.07 | 5335.11 |
| D | 2 | 7.84 | 2.92 | 2 | 30.09 | 14.04 | 41 | 124.77 | 2.04 | 238880.13 | 97579.10 | 87231.83 | 4555.49 |

Table S1a: Estimation of Population having health morbidity due to exposure of SO_2_, NO_2_, and PM_10_ across Mumbai up till 15th August 2020.

Table S1b: Total Estimated Emissions by Sector for 2018 (units – tons/year)

|  | PM_2.5_ | PM_10_ | NO_x_ | SO_2_ |
| --- | --- | --- | --- | --- |
| Transport emissions from road, rail, aviation, and shipping (for coastal cities) | 19.1 | 11.3 | 29.2 | 3.5 |
| Residential emissions from cooking, heating, and lighting activities | 3.0 | 1.8 | 0.1 | 1.1 |
| Industrial emissions from small, medium, and heavy industries (including power generation) | 48.6 | 28.1 | 58.5 | 91.4 |
| Dust emissions from road re-suspension and construction activities | 14.2 | 49.9 | 0.0 | 0.0 |
| Open waste burning emissions | 4.3 | 2.6 | 0.0 | 0.1 |
| Diesel generator set emissions | 5.8 | 3.5 | 9.9 | 0.8 |
| Brick kiln emissions (not included in the industrial emissions) | 4.9 | 2.8 | 2.3 | 3.1 |
|  | 100 | 100 | 100 | 100 |

Source: Calculated by authors based on data provided by urbanemission.info

<https://urbanemissions.info/india-apna/mumbai-india/#:~:text=Air%20pollution%20in%20Mumbai%20is,%2C%20open%20burning%2C%20and%20dust>.


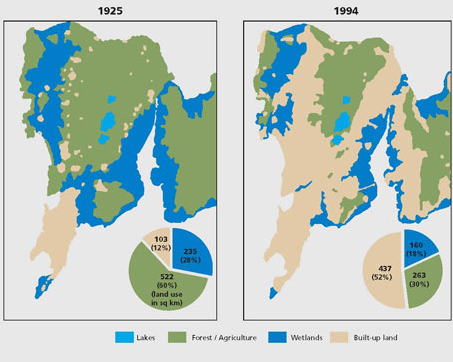


Fig S1a: Wet Lands of Mumba Source: Urban floods: lessons from Jammu & Kashmir. Down to Earth, By Sushmita Sengupta, Thursday 17 September 2015 <https://www.downtoearth.org.in/news/urbanisation/urban-floods-lessons-from-jammu-kashmir-46274>
